# Risk of Concurrent Endometrial Carcinoma in Patients Diagnosed With Atypical Endometrial Hyperplasia: A Retrospective Observational Study

**DOI:** 10.7759/cureus.112062

**Published:** 2026-07-04

**Authors:** Ummul Huda, Y. Srikanth Babu, K. Florence Nightingale, Rasheed Fatima, K. Shilpa, Uzma Tazeen Mohammad, Shweta Keerthi

**Affiliations:** 1 Department of Pathology, Sri Venkata Sai (SVS) Medical College, Mahbubnagar, IND

**Keywords:** atypical endometrial hyperplasia, endometrial carcinoma, endometrial neoplasms, hysterectomy, risk factors

## Abstract

Background

Atypical endometrial hyperplasia (AEH) is a premalignant lesion of the endometrium with a substantial risk of coexisting endometrial carcinoma (EC). Identification of concurrent carcinomas is important for appropriate surgical planning and patient management. This study evaluated the prevalence of concurrent EC in patients diagnosed with AEH and investigated associated clinicopathological risk factors.

Methodology

This retrospective observational study included 260 women with a histopathological diagnosis of AEH based on preoperative endometrial sampling, who subsequently underwent hysterectomy between January 2020 and December 2024. Demographic, clinical, radiological, and pathological data were retrieved from medical records. Continuous variables were compared using the independent-samples t-test, whereas categorical variables were analyzed using the chi-squared test. Binary logistic regression analysis was performed to identify independent predictors of concurrent EC.

Results

The mean age of the study population was 52.4 ± 9.8 years, and the mean body mass index (BMI) was 28.6 ± 5.4 kg/m². Concurrent EC was identified in 98 of the 260 patients, with a prevalence of 37.7%. Patients with concurrent EC were significantly older (56.3 ± 9.6 vs. 50.1 ± 9.3 years; p < 0.001), had higher BMI (30.7 ± 5.4 vs. 27.4 ± 5.1 kg/m²; p < 0.001), and greater endometrial thickness (17.1 ± 6.1 vs. 12.6 ± 4.9 mm; p < 0.001) than those without carcinoma. Postmenopausal status (p = 0.041), diabetes mellitus (p = 0.010), obesity (p = 0.002), abnormal uterine bleeding (p = 0.025), and endometrial thickness ≥15 mm (p < 0.001) were significantly associated with concurrent EC. Endometrioid adenocarcinoma was the predominant subtype, with most tumors classified as grade 1 and International Federation of Gynecology and Obstetrics (FIGO) stage IA. Multivariate analysis identified increasing age (adjusted odds ratio (aOR) = 1.05; p = 0.007), obesity (aOR = 1.94; p=0.027), and endometrial thickness ≥15 mm (aOR = 4.12; p < 0.001) as independent predictors of concurrent carcinoma.

Conclusions

More than one-third of patients with AEH harbored concurrent EC during hysterectomy. Advanced age, obesity, and increased endometrial thickness are significant independent predictors of occult malignancy and may aid in preoperative risk stratification and clinical decision-making.

## Introduction

Endometrial carcinoma (EC) is the most common gynecological malignancy in developed countries and is increasingly being recognized as a major health concern worldwide. Atypical endometrial hyperplasia (AEH), also referred to as endometrial intraepithelial neoplasia (EIN), is considered the principal precursor lesion of endometrioid EC [1,2]. Histologically, AEH is characterized by glandular crowding, architectural complexity, and cytological atypia, reflecting a significant risk of progression to invasive carcinoma [2]. Early identification and appropriate management of this condition are crucial for preventing malignant transformation and improving patient outcomes [3].

The diagnosis of AEH is commonly established through endometrial sampling techniques such as Pipelle biopsy, dilatation and curettage (D&C), or hysteroscopic-guided biopsy [4]. However, these methods sample only a limited portion of the endometrium and may fail to detect coexisting carcinomas. Consequently, a substantial proportion of patients diagnosed preoperatively with AEH have been found to harbor concurrent EC in hysterectomy specimens [5]. The reported rates of concurrent carcinoma vary widely in the literature, ranging from 20% to 50%, highlighting the clinical importance of accurate preoperative risk assessment [6].

Several demographic and clinical factors, including advanced age, obesity, diabetes mellitus, postmenopausal status, and increased endometrial thickness, have been associated with an increased likelihood of concurrent carcinoma [6]. Identifying such predictors can assist clinicians in counseling patients regarding treatment options, determining the extent of surgical staging, and optimizing perioperative planning.

Despite growing evidence, data, particularly regarding clinicopathological predictors and histopathological characteristics of concurrent carcinoma, remain limited. Therefore, evaluating the prevalence and associated risk factors of concurrent EC among women diagnosed with AEH is of considerable clinical significance. The present study aimed to determine the prevalence of concurrent EC in hysterectomy specimens among women diagnosed preoperatively with AEH. Secondary objectives were to evaluate the demographic, clinical, radiological, and pathological factors associated with concurrent carcinoma, characterize the histopathological features of detected malignancies, identify independent predictors using multivariable logistic regression analysis, and assess the diagnostic concordance between preoperative endometrial sampling methods and final hysterectomy specimen diagnoses.

## Materials and methods

Study design and setting

This retrospective observational study was conducted at the Department of Pathology, SVS Medical College, Yenugonda, Mahbubnagar, Telangana, India, after obtaining approval from the Institutional Ethics Committee (IEC/DHR-01/02/(57/113)/2024/047). This study involved a comprehensive review of the hospital medical records and pathology databases. Patients diagnosed with AEH between January 2020 and December 2024 were identified and evaluated. This study was designed to assess the prevalence of concurrent EC in hysterectomy specimens and identify the factors associated with malignant transformation.

Study population

The study population comprised women with a histopathological diagnosis of AEH established through preoperative endometrial sampling. A total of 260 eligible patients, who subsequently underwent hysterectomy and had complete clinical and pathological records, were included in the analysis.

Eligibility criteria

Women aged ≥18 years with a confirmed histopathological diagnosis of AEH or EIN on preoperative endometrial biopsy, D&C, or hysteroscopy-guided biopsy were considered eligible. Only patients who subsequently underwent hysterectomy with available final histopathological examination reports and complete clinical records were included.

Patients with a previously established diagnosis of EC before surgery, incomplete clinical records, unavailable pathology reports, conservative management without hysterectomy, or receiving neoadjuvant hormonal therapy before definitive surgery were excluded from the study.

The diagnosis of AEH/EIN was established according to the World Health Organization (WHO) histopathological criteria based on architectural glandular crowding with cytological atypia. Histopathological slides from preoperative endometrial sampling and corresponding hysterectomy specimens were reviewed by experienced gynecologic pathologists as part of routine institutional practice. Hysterectomy specimens were examined following standard pathology protocols, including gross evaluation of the uterus, serial sectioning of the endometrium, representative sampling of suspicious lesions, and microscopic assessment to determine histological subtype, tumor grade, depth of myometrial invasion, and cervical involvement.

Sample size estimation

Sample size estimation was performed using G*Power software (version 3.1.9.7; Heinrich Heine University, Düsseldorf, Germany). Based on previously published literature reporting a prevalence of concurrent EC of approximately 25% among patients with AEH, a minimum sample size of 235 patients was calculated to achieve 80% statistical power with a two-sided alpha error of 0.05 [7]. Considering a possible 10% rate of incomplete records, the sample size was increased, and a final cohort of 260 patients was included.

Data collection procedure

Data were retrieved from institutional electronic medical records, surgical registers, pathology databases, and archived histopathological reports. Relevant demographic, clinical, radiological, and pathological variables were extracted using a standardized data collection form.

Demographic variables included age, parity, menopausal status, and body mass index (BMI). Clinical variables included presenting symptoms, particularly abnormal uterine bleeding, and associated comorbidities, such as diabetes mellitus and hypertension. Radiological assessment included the measurement of endometrial thickness using transvaginal ultrasonography.

Pathological information included the method of preoperative endometrial sampling, diagnosis of AEH, final hysterectomy specimen diagnosis, histological subtype of carcinoma, tumor grade, myometrial invasion status, and International Federation of Gynecology and Obstetrics (FIGO) stage [8].

Outcome measures

The primary outcome was the prevalence of concurrent EC identified in hysterectomy specimens from patients preoperatively diagnosed with AEH. Secondary outcomes included assessment of demographic and clinicopathological factors associated with concurrent carcinoma, identification of independent risk factors predicting concurrent carcinoma, evaluation of histopathological characteristics of detected carcinomas, and assessment of concordance between preoperative sampling techniques and final hysterectomy diagnoses.

All preoperative endometrial and hysterectomy specimens were processed according to standard pathology protocols. Histological diagnoses were established by gynecological pathologists. Cases of invasive carcinoma in hysterectomy specimens were categorized as concurrent EC. Histological subtype classification, tumor grading, depth of myometrial invasion, and FIGO staging were performed according to the established international guidelines.

Statistical analysis

Statistical analysis was performed using SPSS software (version 26.0; IBM Corp., Armonk, NY, USA). The normality of continuous variables was assessed using the Shapiro-Wilk test. Continuous variables showing a normal distribution were expressed as mean ± standard deviation and compared using the independent-samples t-test. Categorical variables are presented as frequencies and percentages and were analyzed using the chi-squared test or Fisher’s exact test, where appropriate. Variables demonstrating a p-value less than 0.10 on univariate analysis were entered into a multivariate binary logistic regression model to identify independent predictors of concurrent EC. Results were expressed as odds ratios (ORs) and adjusted odds ratios (aORs) with 95% confidence intervals (CIs). Model adequacy was assessed using the Hosmer-Lemeshow goodness-of-fit test and Nagelkerke R² statistic. Statistical significance was set at a p-value <0.05.

## Results

This study included 260 patients with preoperative AEH who subsequently underwent hysterectomy. The mean age was 52.4 ± 9.8 years, and the mean BMI was 28.6 ± 5.4 kg/m². Most patients were postmenopausal (148, 56.9%) and multiparous (212, 81.5%), and presented with abnormal uterine bleeding (244, 93.8%). Hypertension, diabetes mellitus, and obesity were observed in 104 (40.0%), 86 (33.1%), and 96 (36.9%) patients, respectively (Table 1).

**Table 1:** Baseline demographic and clinical characteristics of the study population (N = 260). Continuous variables are presented as mean ± standard deviation; categorical variables are presented as frequency (percentage). BMI = body mass index; D&C = dilatation and curettage

Characteristic	Total (N = 260)
Age (years)	52.4 ± 9.8
BMI (kg/m²)	28.6 ± 5.4
Endometrial thickness (mm)	14.2 ± 5.7
Menopausal status	
Premenopausal	112 (43.1%)
Postmenopausal	148 (56.9%)
Parity	
Nulliparous	48 (18.5%)
Multiparous (≥1)	212 (81.5%)
Abnormal uterine bleeding	244 (93.8%)
Diabetes mellitus	86 (33.1%)
Hypertension	104 (40.0%)
Obesity (BMI ≥ 30 kg/m²)	96 (36.9%)
Method of preoperative sampling	
Endometrial biopsy (Pipelle)	118 (45.4%)
D&C	94 (36.2%)
Hysteroscopic-guided biopsy	48 (18.5%)

Concurrent EC was identified in 98 (37.7%) patients. Patients with concurrent carcinoma were significantly older and had a higher BMI and greater endometrial thickness than those without carcinoma. Postmenopausal status was present in 64 (65.3%) patients with concurrent carcinoma compared with 84 (51.9%) patients without carcinoma. Diabetes mellitus was observed in 42 (42.9%) and 44 (27.2%) patients, whereas obesity was identified in 48 (49.0%) and 48 (29.6%) patients, respectively. Abnormal uterine bleeding was reported in 96 (98.0%) patients with concurrent carcinoma and 148 (91.4%) patients without carcinoma. Endometrial thickness ≥15 mm was observed in 62 (63.3%) patients with concurrent carcinoma compared with 42 (25.9%) patients without carcinoma (Table 2).

**Table 2:** Comparison of clinical and pathological variables between patients with and without concurrent endometrial carcinoma on hysterectomy specimen. Continuous variables are presented as mean ± standard deviation and compared using the independent samples t-test; categorical variables are presented as frequency (percentage) and compared using the chi-square test; *: p < 0.05 was considered statistically significant. EC = endometrial carcinoma; BMI = body mass index

Variable	No EC (n = 162)	Concurrent EC (n = 98)	Test statistic	P-value
Age (years)	50.1 ± 9.3	56.3 ± 9.6	t = −4.87	<0.001*
BMI (kg/m²)	27.4 ± 5.1	30.7 ± 5.4	t = −4.63	<0.001*
Endometrial thickness (mm)	12.6 ± 4.9	17.1 ± 6.1	t = −6.11	<0.001*
Premenopausal	78 (48.1%)	34 (34.7%)	χ² = 4.18	0.041*
Postmenopausal status	84 (51.9%)	64 (65.3%)		
Multiparous (≥1)	138 (85.2%)	74 (75.5%)	χ² = 3.92	0.048*
Nulliparous	24 (14.8%)	24 (24.5%)		
Diabetes mellitus	44 (27.2%)	42 (42.9%)	χ² = 6.68	0.010*
Hypertension	58 (35.8%)	46 (46.9%)	χ² = 3.04	0.081*
Obesity (BMI ≥ 30 kg/m²)	48 (29.6%)	48 (49.0%)	χ² = 9.49	0.002*
Abnormal uterine bleeding	148 (91.4%)	96 (98.0%)	χ² = 5.02*	0.025*
Endometrial thickness ≥15 mm	42 (25.9%)	62 (63.3%)	χ² = 35.8	<0.001*

Among the 98 patients with concurrent carcinoma, endometrioid adenocarcinoma was the predominant histological subtype, occurring in 88 (89.8%) patients. Serous carcinoma was identified in six (6.1%) patients, while clear cell and mucinous carcinomas were observed in two (2.0%) patients. Most tumors were grade 1 (52, 59.1%), followed by grade 2 (26, 29.5%) and grade 3 (10, 11.4%). Myometrial invasion was not observed in 46 (46.9%) patients, superficial invasion in 38 (38.8%), and deep invasion in 14 (14.3%). The majority of carcinomas were diagnosed at FIGO Stage IA (72, 73.5%), followed by Stage IB (14, 14.3%), Stage II (8, 8.2%), and Stage III (4, 4.1%) (Table 3).

**Table 3:** Histopathological characteristics of concurrent endometrial carcinoma identified in hysterectomy specimens (n = 98). Data are presented as frequency (percentage); histological grading was applied only to endometrioid adenocarcinoma; no comparative statistical test was performed, as the table describes histopathological characteristics of concurrent endometrial carcinoma cases. FIGO = International Federation of Gynecology and Obstetrics

Histopathological feature		N (%)
Histological subtype	Endometrioid adenocarcinoma	88 (89.8%)
	Serous carcinoma	6 (6.1%)
	Clear cell carcinoma	2 (2.0%)
	Mucinous carcinoma	2 (2.0%)
FIGO histological grade	Grade 1 (well-differentiated)	52 (59.1%)
	Grade 2 (moderately differentiated)	26 (29.5%)
	Grade 3 (poorly differentiated)	10 (11.4%)
Myometrial invasion	No myometrial invasion (confined to endometrium)	46 (46.9%)
	Superficial invasion (<50% myometrium)	38 (38.8%)
	Deep invasion (≥50% myometrium)	14 (14.3%)
FIGO stage	Stage IA	72 (73.5%)
	Stage IB	14 (14.3%)
	Stage II	8 (8.2%)
	Stage III	4 (4.1%)

Logistic regression analysis demonstrated that increasing age, obesity, and endometrial thickness ≥15 mm were independent predictors of concurrent EC. Endometrial thickness ≥15 mm emerged as the strongest predictor, increasing the likelihood of concurrent carcinoma by more than fourfold (aOR = 4.12; 95% CI = 2.31-7.34; p < 0.001) (Table 4). The model showed good fit (Hosmer-Lemeshow χ² = 7.14, p = 0.521) and explained 31.2% of the variance (Nagelkerke R² = 0.312).

**Table 4:** Univariate and multivariate logistic regression analysis of risk factors for concurrent endometrial carcinoma in patients with atypical endometrial hyperplasia. Univariate and multivariate binary logistic regression analyses were performed to identify predictors of concurrent endometrial carcinoma; results are presented as OR/aOR with 95% confidence intervals; variables with *p < 0.10 in univariate analysis were included in the multivariate model; **: p < 0.05 was considered statistically significant. OR = odds ratio; aOR = adjusted odds ratio; CI = confidence interval; Wald χ² = Wald chi-square statistic

Variable	Univariate analysis		Multivariate analysis		
	OR (95% CI)	P-value	aOR (95% CI)	P-value	Wald χ²
Age (per year increase)	1.07 (1.04–1.11)	<0.001*	1.05 (1.01–1.09)	0.007**	7.28
Postmenopausal status	1.75 (1.03–2.97)	0.038*	1.58 (0.88–2.84)	0.124	2.35
BMI ≥ 30 kg/m²	2.28 (1.33–3.90)	0.003*	1.94 (1.08–3.48)	0.027**	4.88
Diabetes mellitus	2.03 (1.17–3.51)	0.011*	1.72 (0.95–3.12)	0.074**	3.18
Nulliparity	1.87 (0.97–3.60)	0.061*	1.64 (0.82–3.28)	0.162	1.96
Endometrial thickness ≥ 15 mm	4.78 (2.79–8.19)	<0.001*	4.12 (2.31–7.34)	<0.001**	19.64
Abnormal uterine bleeding	5.41 (1.23–23.8)	0.025*	3.86 (0.82–18.2)	0.089	2.89
Hypertension	1.57 (0.94–2.64)	0.085*	1.31 (0.74–2.31)	0.348	0.88

Assessment of diagnostic concordance between preoperative sampling methods and final hysterectomy findings showed upgrade rates to carcinoma of 42 (35.6%) for pipelle biopsy, 36 (38.3%) for D&C, and 20 (41.7%) for hysteroscopy-guided biopsy. No statistically significant differences were observed between the sampling methods (p = 0.634) (Table 5).

**Table 5:** Concordance between preoperative endometrial sampling diagnosis and final hysterectomy specimen findings. Data are presented as frequency (percentage); comparison of sampling method upgrade rates was performed using the chi-square test(χ²); df: degree of freedom; p < 0.05: significant. AEH = atypical endometrial hyperplasia; EC = endometrial carcinoma; D&C = dilatation and curettage

Sampling method	Total, n	AEH confirmed, n (%)	Concurrent EC, n (%)	Test statistics (df)	P-value
Endometrial biopsy (Pipelle)	118	76 (64.4%)	42 (35.6%)	χ² = 0.91 (2)	0.634
D&C	94	58 (61.7%)	36 (38.3%)		
Hysteroscopic-guided biopsy	48	28 (58.3%)	20 (41.7%)		
Overall	260	162 (62.3%)	98 (37.7%)		

## Discussion

This retrospective observational study evaluated the prevalence of concurrent EC among women diagnosed preoperatively with AEH and identified clinicopathological predictors associated with occult malignancy. The principal finding of this study was that 37.7% of patients with AEH harbored concurrent EC in hysterectomy specimens. Furthermore, increasing age, obesity, and endometrial thickness ≥15 mm have emerged as independent predictors of concurrent carcinoma. Most carcinomas are low-grade endometrioid adenocarcinomas that are diagnosed at an early stage.

The observed prevalence of concurrent EC (37.7%) is consistent with previous reports, indicating that approximately one-quarter to one-half of the patients diagnosed with AEH on preoperative sampling may have underlying carcinoma at definitive surgery. Rakha et al. [7] reported concurrent carcinoma rates ranging from 27% to 43% among women undergoing hysterectomy for AEH, emphasizing the limitations of endometrial sampling techniques in excluding invasive disease. Similarly, Trimble et al. [9] reported that the incidence of concurrent carcinoma is 42.6% in women diagnosed with EIN. The prevalence observed in the present study reinforces the concept that AEH represents a high-risk premalignant lesion frequently associated with occult malignancy.

Age was found to be a significant independent predictor of concurrent carcinomas. Women with EC were significantly older than those without EC, and each incremental increase in age increased the likelihood of malignancy. This finding is consistent with those of Lacey et al. [10] and Pennant et al. [11], who demonstrated a strong association between advanced age and malignant progression in women with AEH. Age-related hormonal changes, prolonged estrogen exposure, and accumulation of genetic alterations may contribute to this increased risk.

Obesity has emerged as an independent predictor of EC. Women with a BMI ≥30 kg/m² had nearly twice the risk of carcinoma than non-obese women. Obesity is a well-established risk factor for both AEH and EC, because adipose tissue acts as an extragonadal source of estrogen through the aromatization of androgens. Elevated estrogen levels promote continuous endometrial proliferation and increase the likelihood of malignant transformation. Similar associations have been reported in previous studies, which identified obesity as one of the strongest modifiable risk factors for endometrial carcinogenesis [12,13].

A particularly important finding of the present study was the strong predictive value of increased endometrial thickness. Multivariate analysis showed that patients with endometrial thickness ≥15 mm were more than four times more likely to have concurrent carcinoma. This observation is supported by previous investigations that demonstrated that greater endometrial thickness reflects an increased disease burden and may indicate the presence of invasive carcinoma. Bakos et al. [14] concluded that an endometrial thickness of 6-31 mm is associated with EC and recommended that endometrial thickness >6 mm should be evaluated for endometrial pathology.

The strong predictive value of increased endometrial thickness observed in the present study is consistent with the findings of Heremans et al. [15], who reported that models incorporating endometrial thickness along with clinical and ultrasound variables achieved substantially higher diagnostic accuracy for endometrial malignancy than those incorporating endometrial thickness alone. This highlights the importance of integrating sonographic findings with clinical risk factors when evaluating patients at risk of occult EC.

Consistent with our findings, Li et al. [16] reported that increased endometrial thickness is associated with a higher likelihood of premalignant and malignant endometrial lesions. Their systematic review suggested that thresholds of approximately 11-12 mm may provide better diagnostic performance than lower conventional thresholds, supporting the role of endometrial thickness as an important marker for identifying women at increased risk of underlying EC.

Diabetes mellitus and postmenopausal status showed significant associations with concurrent EC on univariate analysis but lost significance after adjustment for confounding factors [17]. This suggests that their influence may be mediated by age, obesity, and metabolic dysfunctions. Nevertheless, these variables remain clinically relevant because they frequently coexist with the established risk factors in women with AEH.

Histopathological evaluation demonstrated that endometrioid adenocarcinoma accounted for nearly 90% of the concurrent carcinomas. Most tumors were grade 1 lesions confined to the uterus, with approximately three-fourths being classified as FIGO Stage IA. These findings are comparable to those reported by Travaglino et al. [18] and Gültekin et al. [19], who observed that most occult carcinomas detected in patients with AEH were low-grade endometrioid tumors diagnosed at an early stage. The predominance of early-stage disease highlights the importance of hysterectomy as a definitive therapeutic and diagnostic procedure in appropriately selected patients.

The study also demonstrated that the rates of upgrade to carcinoma did not differ significantly between pipelle biopsy, D&C, and hysteroscopic-guided biopsy. Although hysteroscopy-guided biopsy showed a higher upgrade rate, the difference was not statistically significant. Similar findings have been reported in previous studies, suggesting that no currently available sampling method can completely exclude concurrent carcinomas because of the focal and heterogeneous distribution of malignant lesions within the endometrium [20-22].

Clinical implications

Our findings have several important clinical implications. Given that more than one-third of women with AEH were found to have concurrent carcinoma, clinicians should maintain a high index of suspicion when counseling patients regarding management options. Advanced age, obesity, and endometrial thickness ≥15 mm may help identify women at particularly high risk and support decisions regarding definitive surgical treatment and comprehensive intraoperative assessment. The high prevalence of early-stage disease further emphasizes the importance of timely diagnosis and hysterectomy in eligible patients to achieve favorable oncological outcomes.

Limitations

This study has several limitations. First, its retrospective design introduces the possibility of a selection bias and limits its ability to establish causal relationships. Second, the study was conducted at a single tertiary care center, which may restrict the generalizability of the findings. Third, molecular markers and immunohistochemical parameters that may influence malignant progression were not evaluated. Fourth, inter-observer variability in pathological interpretation could not be completely excluded. Additionally, the lack of detailed intraoperative findings, including surgical observations and intraoperative assessment, limited the evaluation of their potential association with concurrent EC. Furthermore, the absence of a centralized pathology review may have introduced diagnostic variability in the classification of AEH/EIN, potentially influencing the estimated prevalence of concurrent EC and associated risk estimates. Finally, long-term oncological outcomes and survival analyses were not available because of the retrospective nature of the study. Future multicenter prospective studies incorporating molecular profiling are warranted to validate these findings and improve risk stratification in patients with AEH.

## Conclusions

Concurrent EC was identified in more than one-third of the patients diagnosed preoperatively with AEH. Age, obesity, and endometrial thickness ≥15 mm were independent predictors of occult malignancy. Most concurrent carcinomas are low-grade endometrioid tumors detected at an early stage, which supports careful risk assessment and definitive surgical management in women with AEH.
